# Assessment of the value of polygenic risk scores in the prevention of disease

**DOI:** 10.1177/09691413251376444

**Published:** 2025-09-10

**Authors:** Aroon D Hingorani

**Affiliations:** Institute of Cardiovascular Science, 4919 University College London, London, UK

**Keywords:** Prediction, screening, risk stratification, polygenic risk score, genomics

## Abstract

It is claimed that polygenic risk scores will transform disease prevention, but a typical polygenic risk score for a common disease only detects 11% of affected individuals at a 5% false positive rate. This level of screening performance is not useful. Claims to the contrary are either due to incorrect interpretation of the data or other influences. Implementation of polygenic risk scores would divert resources from population-wide approaches that address the major disease burden in the average-risk majority to the follow-up of the many false positive results in those designated at high polygenic risk.

## Interest in polygenic risk scores

Genome-wide association studies (GWAS) of common diseases such as myocardial infarction, stroke and cancers have identified tens of thousands of common DNA sequence variants (mainly single nucleotide polymorphisms, SNPs) that influence disease risk.^1^ Attention has been turning to how these discoveries might be used to improve healthcare.^2^

One avenue is to map disease-associated SNPs to the causal genes and encoded proteins to cast light on disease mechanisms and inform the development of new medicines.^3–6^ Another is to apply the new knowledge to disease prediction and prevention through the calculation of polygenic risk scores.^7–9^ A polygenic risk score is calculated for an individual as the weighted sum of independent DNA sequence variants present in their genome that influence the risk of a particular disease. Polygenic risk scores can be readily generated because a person's DNA can be extracted from blood or saliva, and common DNA sequence variation assayed using microarrays or, as costs fall, by whole genome sequencing technology.

## The perceived appeal of polygenic risk scores

Academia, industry and policymakers have expressed enthusiasm for the introduction of polygenic risk scores into healthcare. Academics appear motivated by the desire to find applications for genomic discoveries^10^; industry by the opportunity to sell polygenic risk score services as consumer tests^2,11,12^; and policymakers by the possibility of using polygenic risk scores as a tool for population-wide disease prevention,^13,14^ to alleviate the growing demand on health systems from the management of chronic diseases.

Appealing features of polygenic risk scores include ‘one-off’ measurement at any time from conception, since the DNA sequence of the germline does not change; a single test technology to calculate polygenic risk scores for many different diseases; the low cost of microarray-based genotyping; and the potential for polygenic risk scores to provide information that is independent from non-genetic risk factors.^2^ However, these appealing features are incidental unless polygenic risk scores are useful tests.

## Construction of polygenic risk scores and their screening performance

Researchers construct polygenic risk scores using SNPs whose disease associations surpass a pre-specified statistical significance threshold.^15,16^ Those SNPs that are highly correlated with the most significant (‘lead’) SNP within a region of the genome are either eliminated or down weighted. Depending on the criteria used for SNP selection, a polygenic risk score can include from a few hundred to a few million SNPs.

**Figure 1.**
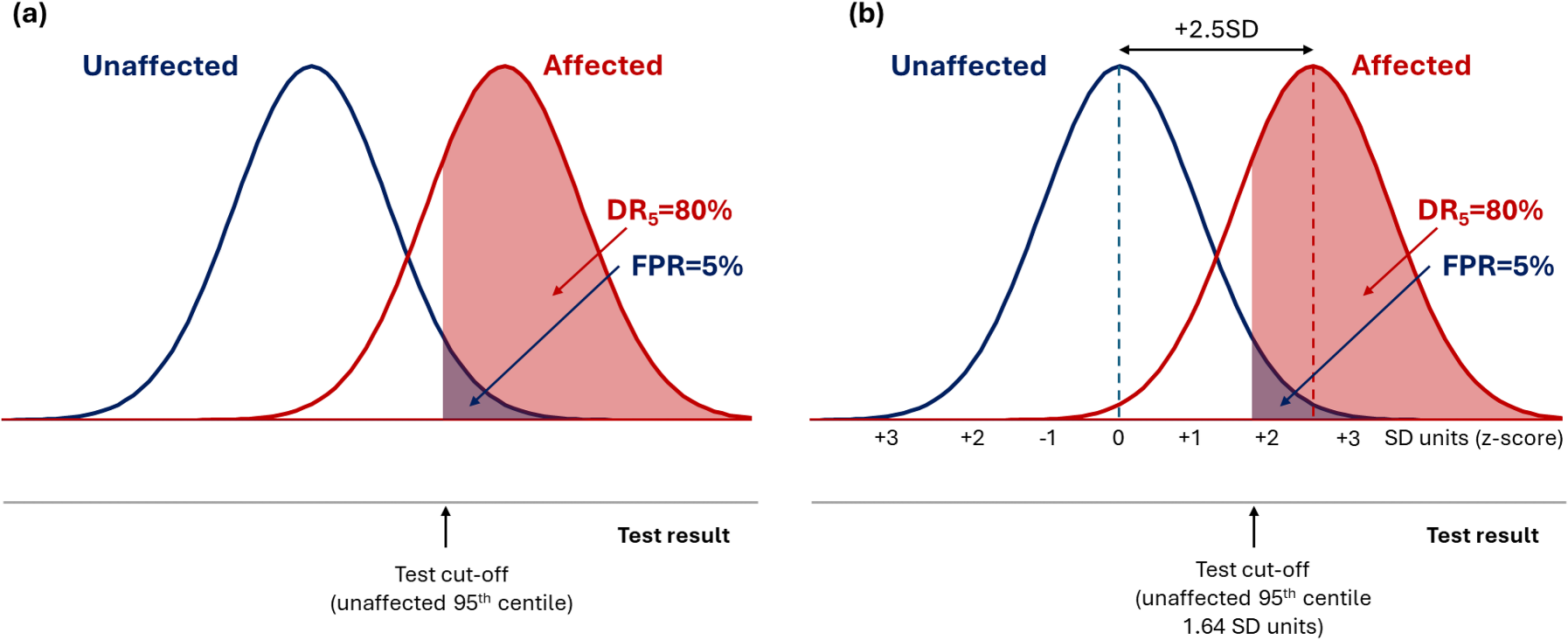
Overlapping distribution of test results in affected and unaffected individuals for a hypothetical screening test with a DR_5_ of 80% (a), showing that the mean value of test results in the two groups are separated by 2.5 SD units (b).

Screening is defined as ‘the systematic application of a test or enquiry to identify individuals at sufficient risk of a specific disorder to benefit from further investigation or direct preventive action, among persons who have not sought medical attention on account of symptoms of that disorder’.^17^ The screening performance of a test is evaluated using the detection rate (DR) and false positive rate (FPR), which are the percentage of people with a test value above a particular cut-off (‘a positive test’) among those who are later affected or unaffected, respectively.^17^ A simple summary measure of screening performance that has been described is the DR for a 5% FPR (DR_5_), that is, a test cut-off at the 95th percentile of the unaffected distribution.^18^ A DR_5_ of 5% is useless, a DR_5_ of 15% is a poor test and a DR_5_ of 80% is a good test, as shown in Figure 1(a). If the distributions of test values in affected and unaffected groups are Gaussian with the same standard deviation (SD), screening performance is determined solely by the difference between the group means expressed in SD units (z-score). Overlapping distributions producing a DR_5_ of 80% are separated by +2.5 SD units (Figure 1(b)).

**Figure 2.**
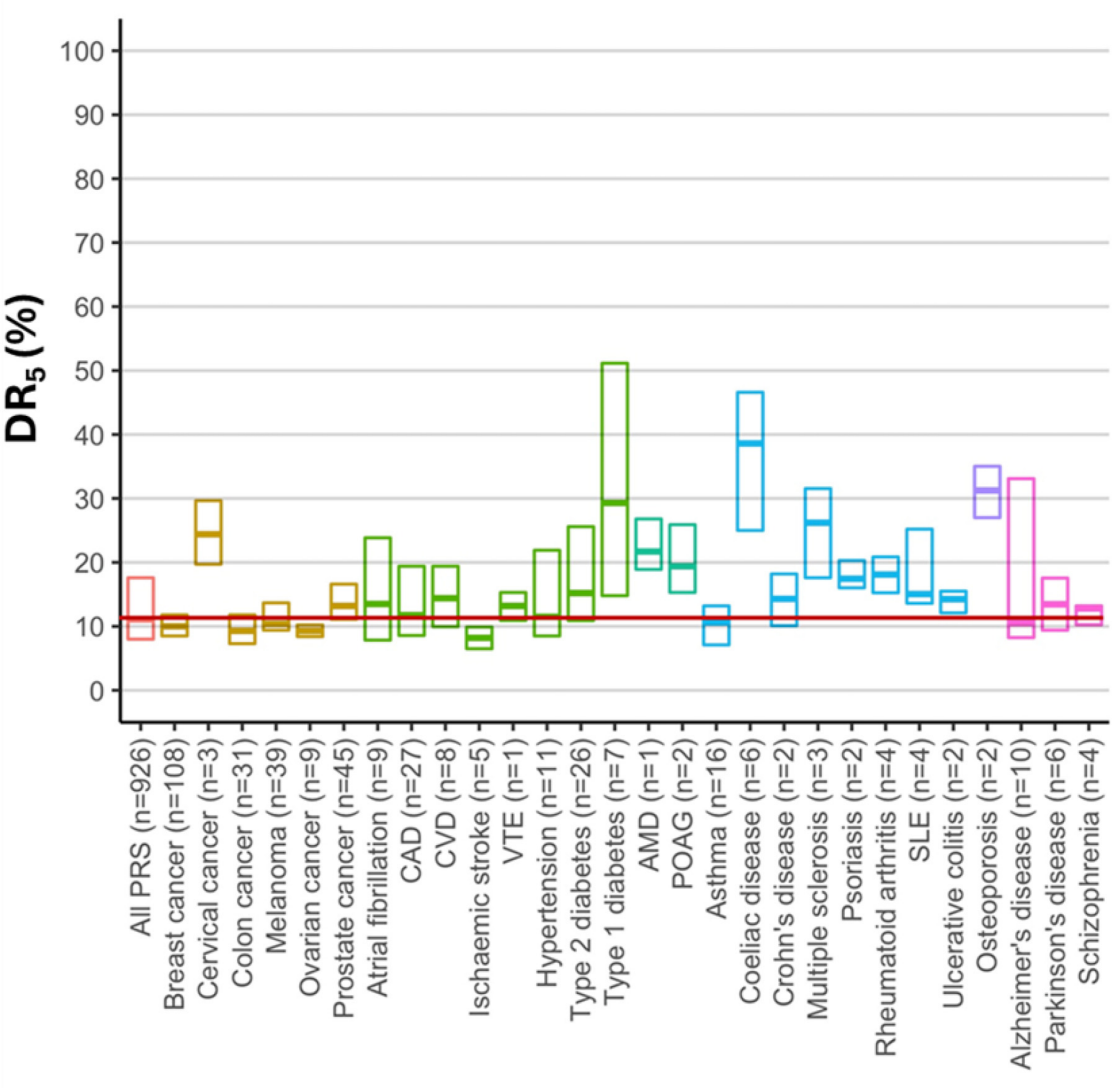
Screening performance of polygenic risk scores for 28 common diseases (adapted from ref^19^). The scores analysed were deposited in the Polygenic Score Catalog up to April 2022. The horizontal line within each box is the median estimated DR_5_ (%) and the limits of each box represent interquartile range; n = number of studies for each disease.

**Figure 3.**
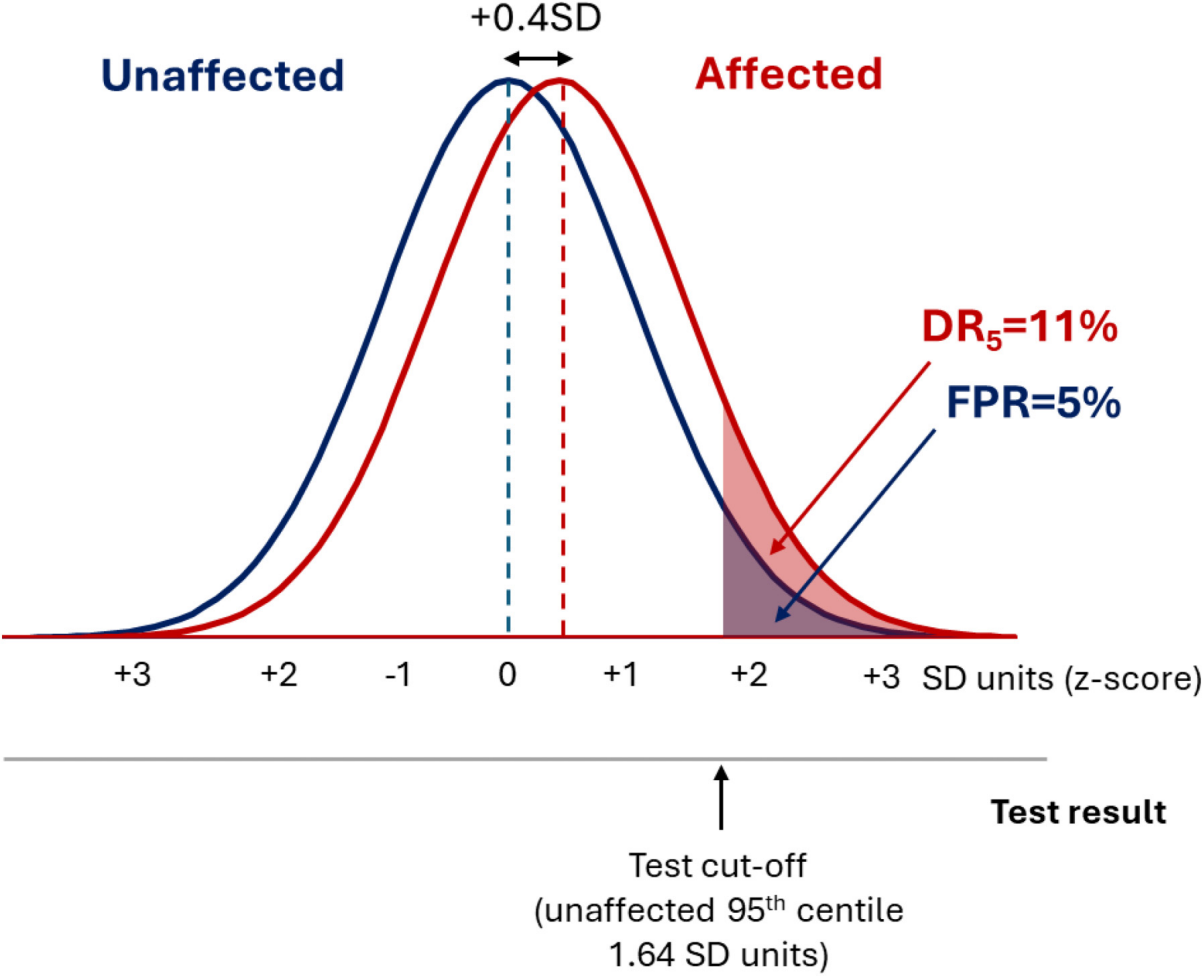
Polygenic risk scores have a Gaussian distribution, with the distribution in affected individuals having the same standard deviation but being shifted to the right with respect to the unaffected individuals. In the typical example shown, the mean value in the affected group lies 0.4SD units to the right of the mean value in the affected group, which produces a DR_5_ of 11%.

Polygenic risk scores display a Gaussian distribution with the same SD in affected and unaffected groups.^19^ Figure 2 shows that the median DR_5_ value for polygenic risk scores for 28 common diseases deposited in the PGS Catalog as of April 2022 was 11%, indicative of poor screening.^19^ Figure 3 shows that a DR_5_ of 11% corresponds to a difference in affected and unaffected group distributions of +0.4SD units, much less than the +2.5SD unit separation seen for a good screening test with a DR_5_ of 80%. A polygenic risk score with a DR_5_ of 11% misses 89% of affected individuals. When applied to a population with a background odds of disease of 1: 9 (10% risk), such a score yields an odds of becoming affected given a positive result of 0.11 × 1 : 0.05 × 9 = 1 : 4 (a positive predictive value of 20%), which is too low to be useful. It is unlikely that more recent polygenic risk scores deposited in the PGS Catalog data after April 2022 will materially alter estimates of screening performance because the major risk loci of largest effect are discovered early^20^ and, as exemplified below for coronary artery disease (CAD), results of older and newer polygenic risk score studies provide similar results.

### Misleading screening performance measures

**Figure 4.**
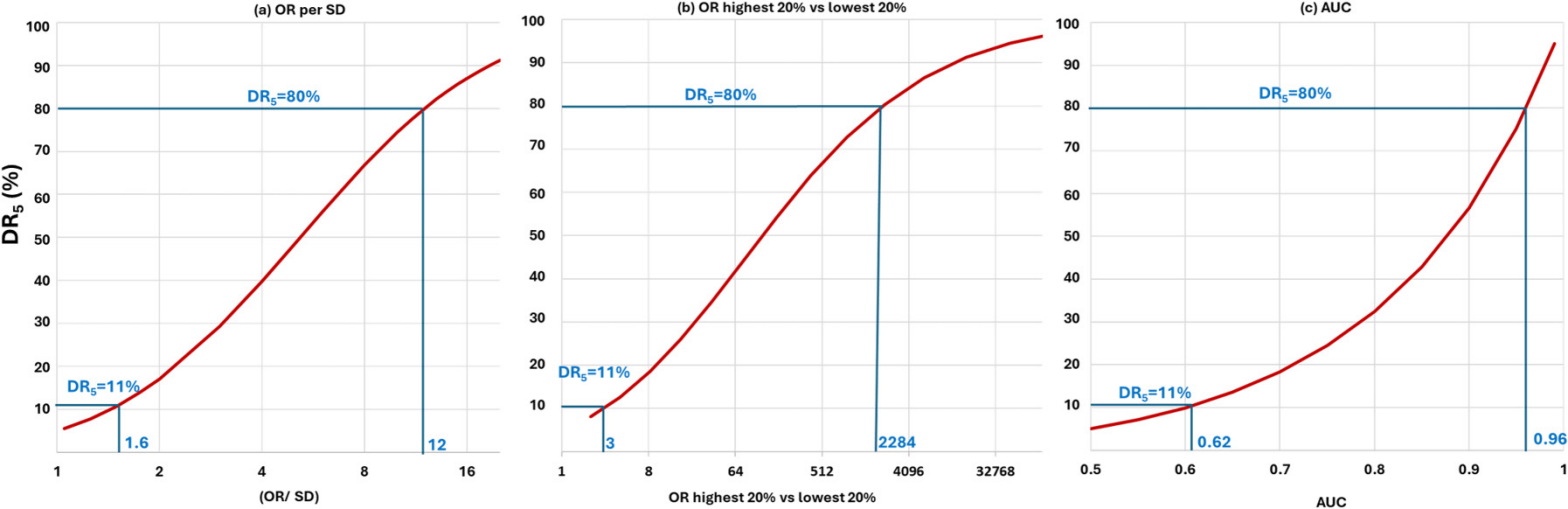
Relationship between the DR_5_ and commonly reported polygenic risk score performance measures: (a) OR per SD; (b) OR comparing the highest and lowest 20% of a polygenic risk score distribution; and (c) AUC. Values of each performance measure are shown that correspond to a DR_5_ of 11% (the median DR_5_ value of polygenic risk scores in the PGS Catalog as of April 2022) and, as a benchmark, values that correspond to a DR_5_ of 80%.

The poor performance of polygenic risk scores in screening is not widely appreciated because the DR and FPR are almost never disclosed in research papers, and nor do they appear in the Polygenic Score Catalog, a regularly updated repository of polygenic risk scores.^21^ Instead, many papers report (a) the odds ratio (OR) for a 1-SD increment in the score (OR/SD), (b) the OR comparing the highest with the lowest 20% of the polygenic risk score distribution, or (c) the area under the receiver operating characteristic curve (AUC). For a test with a DR_5_ of 80%, which represents a good screening performance, the corresponding values for these measures are (a) 12, (b) 2284 and (c) 0.96, respectively (as shown in Figure 4). The screening performance of polygenic risk scores falls well short of this. The corresponding values of these measures for polygenic scores with a median DR_5_ of 11% are (a) 1.6, (b) 3 and (c) 0.62, respectively (as also shown in Figure 4).

**Figure 5.**
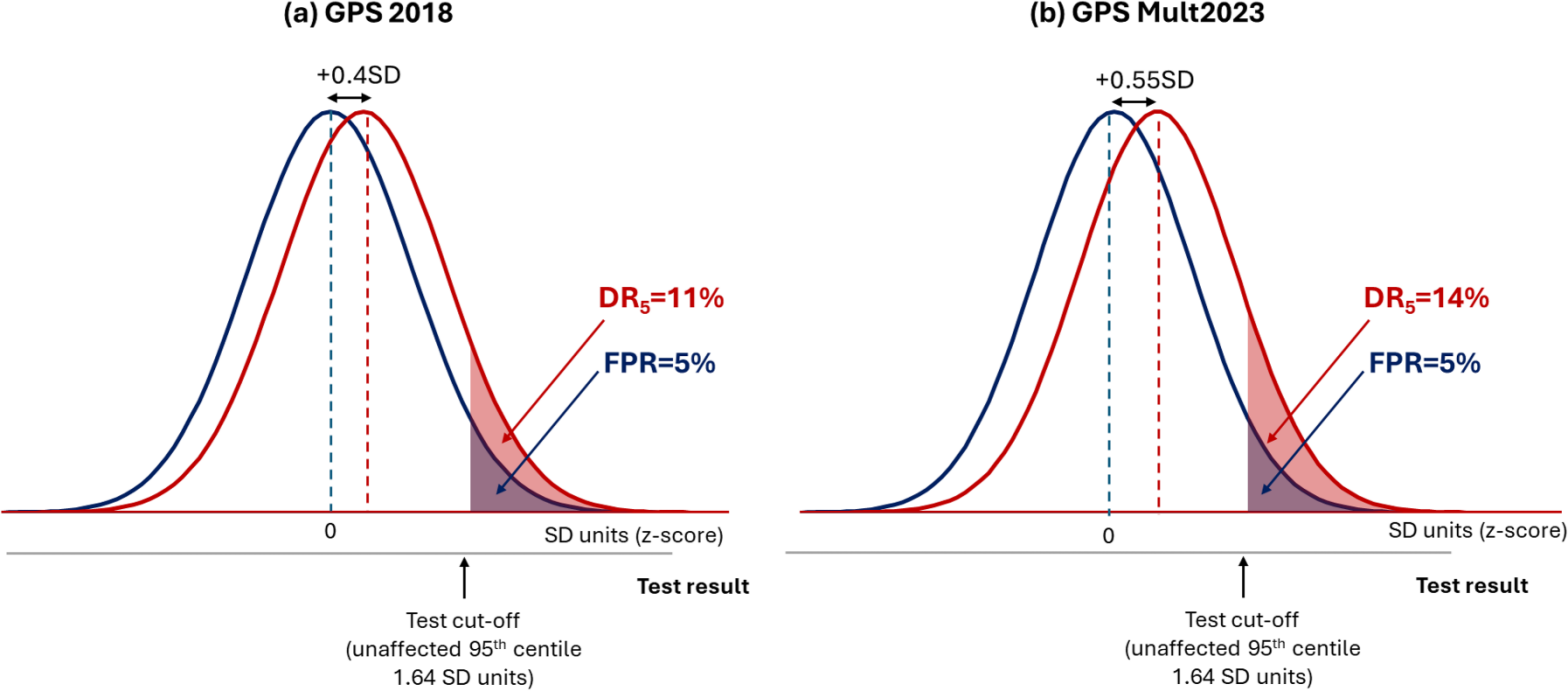
Negligible improvement in the performance of polygenic risk scores for coronary artery disease between 2018 and 2023. (a) DR_5_ = 11% for GPS 2018 based on the reported OR/SD = 1.49 and (b) DR_5_ = 14% for GPS Mult 2023 based on the reported OR/SD = 1.73.^22^

Use of misleading performance measures has also led to misinterpretation of the scale of any performance improvement when a polygenic risk score is updated. For example, a polygenic risk score for CAD reported in 2023 (referred to here as GPS Mult2023) used information from the latest GWAS of >269,000 cases and >1,178,000 controls arising from multiple ancestries, and incorporated SNPs for 10 CAD risk factors including blood pressure and LDL-cholesterol.^22^ The authors concluded that this 2023 score with an OR/SD of 1.73 exhibited a ‘significant’ improvement over their previously published 2018 score which had an OR/SD of 1.49.^23^ Figure 5 shows this difference equates to a trivial improvement in the DR_5_ from 11% to 14%. Similarly, Genomics Ltd reported that their ‘enhanced’ polygenic risk scores for common diseases outperformed almost all comparator scores.^24^ But the reported improvements in OR/SD values equated to negligible improvement in the DR_5_ of around 2%, for example from 8% to 10% for cardiovascular disease, and 13% to 15% for breast cancer.

### Misleading graphical displays of screening performance

**Figure 6.**
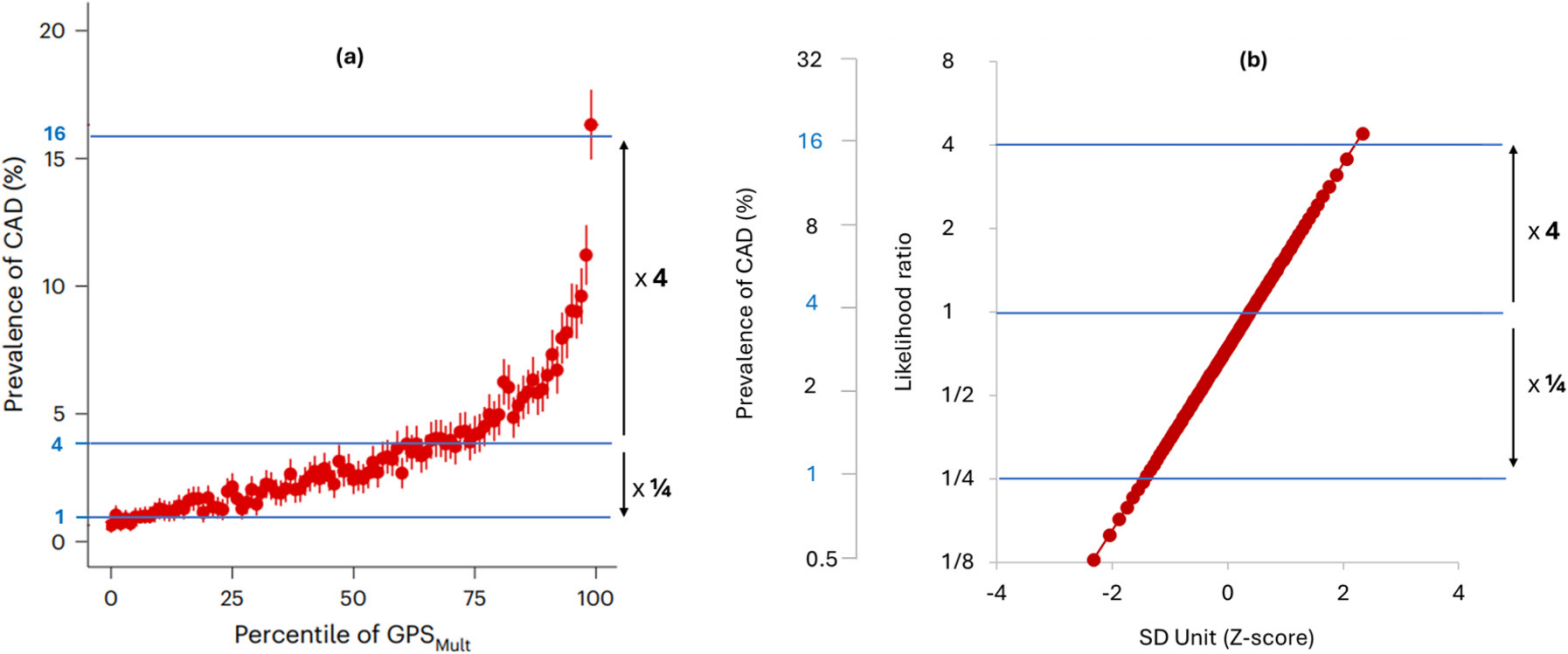
Performance of the same polygenic risk score (GPS Mult2023) depicted using different choices for axes and scales. Image (a) is adapted from the original publication^22^ and (b) The likelihood ratio and prevalence of CAD using a logarithmic scale on the vertical axis, and the polygenic risk score centile using an SD unit (Z-score) scale for the horizontal axis.

Common graphical displays seen in research papers exaggerate the screening performance of polygenic risk scores. Figure 6(a) (taken from Patel et al.^22^) shows a common display type in which the risk of CAD is plotted on the vertical axis using an arithmetic scale, with the percentile of the polygenic risk score GPS Mult2023 on the horizontal scale. Use of an arithmetic *y*-axis scale produces a sharp uptick in CAD risk above the 95^th^ centile of the polygenic risk score distribution, as if identifying a distinct high-risk group. It is claimed that this apparently distinct group have a CAD risk as high as carriers of a mutation causing monogenic familial hypercholesterolaemia (FH).^22,23^

Figure 6(b) also displays the performance of GPS Mult2023 but using the likelihood ratio on a logarithmic (doubling) scale for the vertical axis and an SD (z-score) scale for the horizontal axis. Multiplying the likelihood ratio by the background population odds of disease and converting to the risk scale returns the corresponding disease risk (as shown in Figure 6(b)). This plot produces a straight-line relationship without any indication of an uptick in risk. A logarithmic vertical axis scale is appropriate because disease risks that are, for example, 4 and ¼ times the population average should be located equidistant from the average population risk. The straight-line relationship shows that the same arithmetic difference in the polygenic risk score produces the same proportional difference in disease risk across the whole range of values without a threshold: a log-linear relationship. The apparently sharp rise in risk in the original plot is seen to be an artefact of the choice of axis and scaling: an arithmetic risk scale compresses values <1 and expands values above 1. People in the top 5% of this polygenic risk score distribution do have a 3-fold higher risk of CAD compared to the remainder of the population, but this is much less than the 15-fold relative risk of CAD among FH mutation carriers up to age 50. The claim of a risk equivalence applies only to those FH mutation carriers who have survived beyond age 60 without a prior CAD event.^25,26^

## Performance of polygenic risk scores in stratification and sequential screening

**Figure 7.**
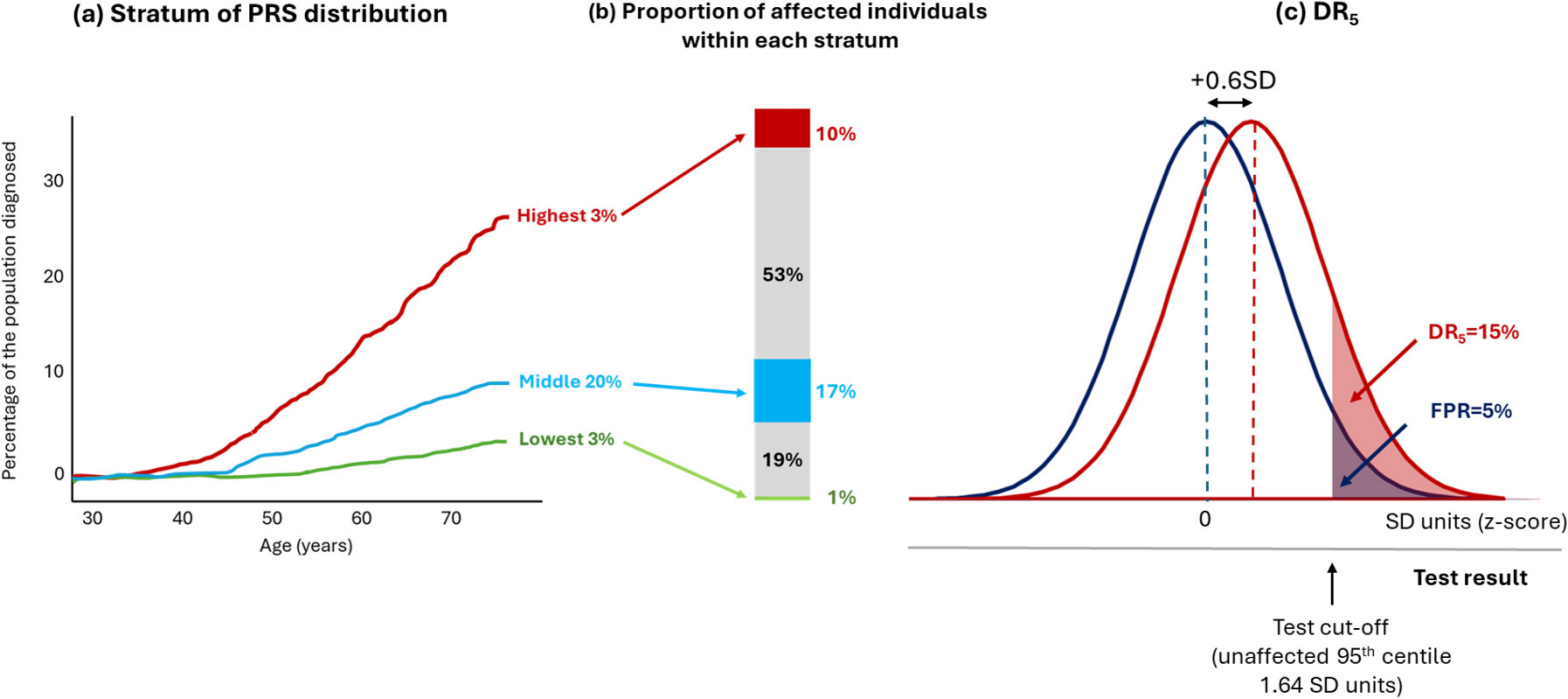
Proportion of affected individuals by strata of a breast cancer polygenic risk score. (a) The incidence of diagnosed breast cancer by age for the highest and lowest 3% of the polygenic risk score distribution, with the middle 20% of the distribution used as a reference group (modified and redrawn from a figure in ref^28^). (b) The proportion of affected individuals in each of the strata depicted and omitted from (a). (c) The overlapping distributions and DR_5_ value for the same breast cancer polygenic risk score.

Stratification is a special case of screening which involves using more than one test cut-off to segment a population into groups of differing risk.^27^ Such information might help tailor the type or intensity of a preventive intervention, or the timing or frequency of a definitive but costly screening test. Figure 7(a), which is based on a figure on the Genomics Ltd website,^12^ shows a typically used display intended to illustrate effective population stratification for a breast cancer polygenic risk score developed by the company.^28^ The plot shows breast cancer incidence by age for the highest and lowest 3% of a breast cancer polygenic risk score distribution, with the middle 20% of the distribution being used as a reference.

This plot omits information on 74% (100 − [3 + 3 + 20] %) of the population. It also provides no information on the proportion of affected individuals in each polygenic risk score category. Figure 7(b) uses the published performance of the Genomics Ltd breast cancer polygenic risk score in Europeans^29^ to show that the middle 20% of the distribution (the reference group) contribute 17% of affected individuals, more than the highest and lowest 3% of the distribution, which contribute 10% and 1% of affected individuals, respectively. Those with a polygenic risk score between the lowest 3% and the middle 20% contribute 19% of affected individuals, and those with a polygenic risk score between the middle 20% and highest 3% contribute 53% of affected individuals. Neither of the latter two groups, which contribute most of the cases, was shown in the original plot.

Where a risk factor has a Gaussian distribution and displays a log-linear relationship with disease risk, more cases arise among the majority with near average risk factor values than among the few with more extreme values – the ‘prevention paradox’.^30,31^ This is made clear in Figure 7(c), which shows that the overlapping distributions for affected and unaffected individuals for the same breast cancer polygenic risk score, corresponding to a DR_5_ of 15. This re-analysis shows why polygenic risk scores such as this perform poorly in stratification as well as in screening: high-risk groups contain more false than true positives and the greater proportion of affected individuals occur among those designated as average risk.

The plot in Figure 7(a) has also been used to argue that women with a high polygenic risk score should be offered mammographic screening a decade earlier than is routine because they achieve a similar breast cancer risk at around age 40 as an average woman at age 50, the age at which mammography is currently offered to all women. Table 1 shows data from Huntley et al., who used UK demographic and cause-specific cancer incidence data to estimate the outcome of breast cancer screening in younger women (aged 40–49) who have a breast cancer polygenic risk score in the highest 20% of the distribution.^32^ There are around 4 million women in the this decade, below the current screening age of 50, among whom 7533 breast cancers were estimated to arise annually with 693 breast cancer deaths. All 4 million women would need to be genotyped to identify the 900,000 or so in the top 20% of the polygenic risk score distribution. Huntley et al. estimated that 2811 cancers would occur in this group of which 1968 would be detected at mammography with an estimated 102 deaths averted by subsequent intervention.^32^ However, Huntley et al. also showed that an alternative approach of simply screening the highest 20% by age (in effect reducing the universal screening age from 50 to 48 years) would detect a similar number of additional breast cancers and avert a similar number of deaths (Table 1). This simpler alternative removes the need to genotype, analyse and interpret polygenic risk scores from 4 million women while achieving almost the same additional benefit.

**Table 1.** Comparison of top PRS quintile-based or top-age quintile-based sequential breast cancer screening for women aged 40–49.

	Population offered screening	
Age	40–49	
Number	4,369,703	
Breast cancers annually	7533	
Breast cancer deaths	694	

Mammography offered to	PRS top quintile	Oldest quintile (ages 48–49)
Number	873,941	937,850
Cancers in the top quintile	2811	2198
Cancers detected	1968	1538
Deaths averted (intervention)	102	80

Data are taken from Huntley et al.^32^

## Polygenic risk scores used in conjunction with non-genetic screening tests

Proponents argue that polygenic risk scores should not be used as stand-alone tests but should be incorporated into risk models that include clinical variables, as part of established screening pathways.

A pilot study in the UK of 836 people aged 45–64 years attending an NHS Health Check, funded by Genomics Ltd, evaluated the feasibility of adding a polygenic risk score to the QRISK model based on conventional cardiovascular risk factors that is currently used in primary prevention.^33^ Re-analysis of the data in this publication shows that inclusion of a polygenic risk score in the QRISK model made no difference to statin eligibility for 90% of participants evaluated. In 5% of the participants, 10-year risk of cardiovascular disease went from below 10% using QRISK alone to above 10% after the addition of a polygenic risk score, rendering this group eligible for statin treatment. However, the up classification of statin eligibility in this group was exactly offset by a downgrading of risk in the remaining 5% of participants, resulting in no overall change in the proportion of people eligible for statins from the addition of a polygenic risk score to QRISK.

Sun et al. claimed that adding a polygenic risk score to conventional cardiovascular risk factors produced worthwhile improvement in the prediction and prevention of CAD and stroke.^34^ However, this is not borne out by a re-analysis of their published data.^19^ In Sun et al., a conventional risk factor model with a 10% 10-year risk cut-off detected 60% of those later affected by CAD or stroke at a 24% FPR (DR_24_ = 60%). The addition of a polygenic risk score produced a negligible increase in DR to 61% at a 23% FPR (DR_23_ = 61%). Assuming statins were prescribed to all those with a 10-year risk exceeding 10%, 100% adherence to treatment, and adopting the authors’ assumption that statins reduce the risk of CAD and stroke by 20%, 974 events would be prevented per 100,000 people screened using a model based on conventional risk factors together with polygenic risk scores instead of 957 using a conventional risk factor model with no genetic information; a gain of 17 cases. This gives a number needed-to-genotype to prevent one additional event of 5882. A re-analysis of similar data published by Genomics Ltd revealed consistent findings.^35^ The QRISK3 model based on conventional risk factors, using the same 10% 10-year risk cut-off as used by Sun et al., detected 81% of affected individuals at a 42% FPR (DR_42_ = 81%) in UK Biobank. Addition of their polygenic risk score to the model detected 84% of affected individuals with a 41% FPR (DR_41_ = 84%). With the assumption that statins reduce CAD and stroke events by 20%, the number needed-to-genotype to prevent one additional event based on this study was 8879.

These results should not be surprising. An independent risk factor that performs poorly on its own will also perform poorly when incorporated into a risk model together with the other risk factors. A simpler approach to cardiovascular prevention is to use age as the sole screening test. Age is the major determinant of CAD and stroke risk and performs about as well as multi-factor risk models that include age.^36^ Offering statins and low-dose blood pressure lowering medications in combination to all those without contraindications above the age of 50 has been estimated to prevent 60% of heart attacks and strokes assuming complete adherence, without the requirement for risk assessment or genetic testing.^37,38^

## Polygenic risk scores in individual risk prediction and as direct-to-consumer tests

Some cohort studies (e.g. the EMERGE Consortium in the US and the Our Future Health Study^39^ in the UK) aim to return polygenic risk score results to study participants. The EMERGE Consortium returns results for eight diseases (asthma, atrial fibrillation, breast cancer in women and prostate cancer in men, coronary heart disease, chronic kidney disease, and type 1 and type 2 diabetes). For six of eight diseases, the EMERGE Consortium genetic report designates a participant as ‘high’ or ‘average’ risk.^40^ The proportion of the population designated high risk differs by disease. For example, 10% of the population are designated high risk for prostate cancer with a 4-fold higher risk than the remaining 90% of the population who are labelled ‘average’ risk. This equates to a test with a 26% DR for a 10% FPR, very poor discrimination. The EMERGE consortium designates only 2% of the population as high risk for type 2 diabetes based on a 4-fold risk compared to the remainder of the population which equates to a DR of 6% for a 2% FPR, missing 94% of cases. No absolute measure of risk is provided to participants for these conditions. Absolute risk estimates are provided to participants only for breast cancer and coronary heart disease as part of an integrated risk model including non-genetic risk factors.

Companies are developing polygenic risk scores as a direct-to-consumer test.^11^ In the UK, the House of Commons Science and Technology Committee 2021 report entitled *Direct-to-Consumer Genetic Testing*^41^ called for greater regulation of such tests, strict clinical performance requirements, independent validation of test performance, and closer scrutiny of the information provided by companies to consumers. The Committee also raised concerns about increased pressure on the publicly funded National Health Service (NHS) that might be generated by consumers seeking advice and follow-up of private sector tests. Unfounded claims of the accuracy and benefits of direct-to-consumer tests in general have been highlighted by others.^42,43^

Since the publication of the House of Commons Committee report, a further concern has emerged about the apparent instability of a polygenic risk score result for an individual. Abramovitz and colleagues tested 46 different polygenic risk scores for CAD in 170,000 participants from the All of Us study.^44^ They found that all the scores tested performed consistently poorly at group level (DR_5_ values were in the range 6–10%), but highly inconsistently for any individual. The same individual could have a CAD polygenic risk score result as far apart as the 5^th^ or 95^th^ centile, depending on the polygenic risk score used. The source of this inconsistency is unresolved but may relate to the practice of including millions of genetic variants in polygenic risk scores, most with tiny effect sizes and statistical significance below the threshold applied in GWAS to declare aetiological association. The inclusion or exclusion of such SNPs may be close to random, adding more noise than signal that varies from score to score. Whatever the explanation for the within-individual variability of results from different polygenic risk scores for the same disease, the observation undermines confidence in the use of polygenic risk scores for individual risk assessment.

## Reflections on the state of the field

Many thousands of research papers have been published on polygenic risk scores in prediction of common diseases. Almost all report the same misleading measures and graphical displays of performance and mistakenly conclude that polygenic risk scores represent an advance in disease prevention.

It is over several decades that the correct analysis of potential screening tests has been described^45^ and over 5 years since this was first applied to polygenic risk scores showing their poor screening performance.^46^ ORs comparing top and bottom quintile groups of 10, 100, 1000 and 10,000 yield DR_5_ values of about 20%, 50%, 75% and 90% respectively. Polygenic risk scores do not even achieve an OR of 10. The freely available Risk Screening Converter produces DR and FPR values from OR comparisons for quintile groups, the OR/SD and AUC.^47,48^ Yet this knowledge and this resource have largely been ignored by researchers in the field.

Worse, there is evidence of a failure to cite key papers (e.g. references^46,49–53^) and a reluctance to publish papers that question the value of polygenic risk scores in prevention. The *BMJ Medicine* paper^19^ that demonstrated the poor performance of over 900 polygenic risk scores for 300 diseases in screening, risk stratification and individual risk prediction has an Altmetric attention score of 1296 (in the top 5% of all research articles) but was rejected by seven journals before eventual publication nearly two years after initial submission. Five rejections were without peer review because the editors did not consider the paper to be a ‘sufficient advance’. Reviewers and editors revealed misconceptions and contradictions in their feedback. The use of polygenic risk scores clearly meets the definition of screening. However, reviewers ‘raised concerns about the premise that polygenic risk scores are screening tests’. In endorsing the use of the AUC (but not the DR_5_) as an appropriate performance measure, reviewers appeared to be unaware of the contradiction that the AUC is a measure of screening performance, albeit an unhelpful one, and that the DR_5_ is one data point on the ROC curve which is used to derive the AUC. After publication of the *BMJ Medicine* paper,^19^ social media was used in an attempt to diminish the importance of the findings.

It is difficult to escape the conclusion that many researchers are choosing to ignore the evidence that polygenic risk scores lack value in screening and disease prediction, even acting as if such evidence did not exist, and that journal editors and reviewers are playing a part in this.

These actions have had unwarranted consequences. Opinion leaders and expert groups are now arguing for implementation of polygenic risk scores for disease prevention^9,10^; over 20 commercial testing and software service providers have been established to sell polygenic risk score tests to consumers and healthcare providers; and some life and health insurers now offer polygenic risk scores to their customers.^9,54,55^ In an example of policy leapfrogging evidence,^14^ *Fit for the Future,* the 10-year Health Plan for England, seeks to ‘implement universal newborn genomic testing and population based polygenic risk scoring alongside other emerging diagnostic tools’. It is claimed this will enable ‘early identification and intervention for individuals at high risk of developing common diseases’. All these initiatives are based on the false premise that polygenic risk scores are of benefit in screening, risk stratification and disease prediction despite overwhelming evidence to the contrary.

Several actions are now required. All those engaged in polygenic risk score research should reflect on their responsibilities to the scientific endeavour, when writing, reviewing and publishing papers, or commenting on social media. Policymakers should impose tighter regulation of commercial providers of polygenic risk scores to protect consumers from purchasing unhelpful or misleading genetic tests. Follow-up of ‘high-risk’ (largely false positive) results should be made the responsibility of the companies selling such tests, not already stretched public healthcare systems. Health systems should use the same established structures for evaluating polygenic risk score performance as used for non-genetic screening tests and hold them to the same standards. There should be renewed focus on simple, ‘low-tech’, untargeted, population-wide approaches to prevention that address the greater burden of disease among the average-risk majority.

## Conclusion

An assessment of the evidence leads to the inescapable conclusion that polygenic risk scores are not of value in the prevention of disease.
